# Testes genéticos de ancestralidade: entre raça, corpo e genoma

**DOI:** 10.1590/S0104-59702025000100002

**Published:** 2025-02-14

**Authors:** Angelo Tenfen Nicoladeli

**Affiliations:** i Doutorando, Casa de Oswaldo Cruz/Fiocruz. Rio de Janeiro – RJ – Brasil. angelonicoladeli@hotmail.com

Sarah Abel é pesquisadora do Instituto de Pesquisas Filosóficas da Universidad Nacional Autónoma de México. Possui formação em Letras e mestrado em Estudos Latino-americanos pela Universidade de Cambridge, tendo concluído seu doutorado na École des Hautes Études en Sciences Sociales, em Paris, com tese sobre testes de ancestralidade genética nos EUA e no Brasil. Abel realizou extenso trabalho de campo em vários países do continente americano, explorando temas como raça, etnia e produção científica, e sua pesquisa foi apoiada pela Comissão Europeia e pela Academia Britânica.

No livro *Permanent markers* (Abel, 2022), a autora aborda a relação entre testes de DNA, raça e identidade, criticando a visão simplista de que o DNA define raça de forma definitiva. Ela destaca como a mídia e a ciência podem reforçar estereótipos e reduzir a ancestralidade de forma simplista, além de questionar a confiabilidade dos testes e manifestar preocupações éticas com as práticas da indústria da área. O livro examina o impacto desses testes nos EUA e no Brasil, reconhecendo seu potencial para desafiar estereótipos e conectar pessoas com suas raízes africanas, ao mesmo tempo que alerta para as complexidades da identidade e para os riscos de discriminação.

No primeiro capítulo, a obra compara os testes de DNA de ancestralidade nos EUA e no Brasil, explorando os documentários *African American Lives* e *Raízes Afro-brasileiras*. A autora destaca como a descoberta da ancestralidade africana é interpretada de modos distintos em cada país. Nos EUA, busca-se por origens étnicas específicas, enquanto no Brasil enfatiza-se a mistura genética e a identidade racial. A partir daí, Abel questiona a neutralidade desses produtos, destacando o modo como fatores históricos, sociais e culturais moldam suas interpretações. Ao analisar a mestiçagem no Brasil e o orgulho negro nos EUA, ela mostra como essas ideias influenciam a percepção da ancestralidade revelada pelo DNA, ressaltando a diversidade de interpretações e a necessidade de questionar a certeza científica sobre raça e identidade.

O capítulo dois analisa os desafios éticos que os geneticistas enfrentam na indústria dos testes. A partir de estudos de caso, como o da empresa African Ancestry e o projeto brasileiro Candela, Abel revela problemas como a falta de transparência, a exclusão de marcadores genéticos e a simplificação excessiva dos dados. Cientistas de diferentes países expressam suas preocupações com a perpetuação de visões racializadas e a redução excessiva das informações genéticas. O capítulo conclui que a interpretação dos resultados deve ser feita com cautela para evitar reforçar estereótipos raciais e promover uma compreensão mais ampla da identidade humana.

O terceiro capítulo critica a indústria que lida com essas técnicas genéticas – argumentando que o modo pelo qual ela as tem empregado promove a comercialização da identidade étnica –, bem como as implicações éticas e políticas envolvidas. A autora destaca a falta de objetividade nos resultados, influenciada pela busca do lucro e pela priorização da satisfação dos clientes, além da composição enviesada dos bancos de dados das empresas. O texto discute questões como cidadania biológica, privacidade e racialização, convidando à reflexão sobre como essas tecnologias moldam nossa compreensão de identidade e ancestralidade.

O capítulo quatro aborda a influência dos testes de ancestralidade na identidade racial nos EUA e no Brasil. Revela que os resultados raramente alteraram de forma drástica a identidade dos participantes entrevistados. No Brasil, onde a identidade racial está mais relacionada à cor da pele, houve pouco interesse em “branquear” a identidade. Nos EUA, alguns afro-americanos viram os resultados como validação de identidades mistas, desafiando a “regra da gota”. Os testes também impactaram a adesão a identidades multirraciais e mestiças, mesmo em uma sociedade marcada por dicotomias raciais, como a norte-americana. O capítulo reconhece as limitações dos estudos, mas sugere que os testes têm uma relação complexa com a identidade racial, fornecendo novas informações que são integradas às compreensões existentes de raça e etnia.

No quinto capítulo, a autora examina como afro-americanos e afro-brasileiros buscam suas identidades ancestrais por meio de testes e pesquisa genealógica. Ela critica a distinção inflexível entre raça e etnia, reconhecendo o valor desses métodos, sobretudo devido ao apagamento histórico das identidades afrodescendentes. Ao questionar a ideia de identidades ancestrais “autênticas”, a autora enfatiza a importância de interpretar os resultados de forma equilibrada, considerando os contextos históricos, sociais e culturais mais amplos.

No epílogo, Abel discute o impacto social dos testes de ancestralidade, alertando sobre o perigo de depender exclusivamente da genética para compreender questões raciais e étnicas. A autora critica a visão de empresas como a AncestryDNA de que esses testes combatem, por si só, o racismo, destacando a importância de ações políticas e educativas contínuas para enfrentar as desigualdades raciais. Ela também adverte sobre o uso dessas tecnologias por grupos supremacistas brancos e enfatiza a necessidade de uma análise contextual dos resultados dos testes genéticos, reconhecendo os riscos de simplificação e instrumentalização desses dados.

*Permanent markers* pode nos remeter ao clássico trabalho de Evelyn Fox Keller (2002) e nos ajudar a perceber que seu alerta continua atual: precisamos estar atentos para os motivos pelos quais o discurso sobre os genes vem sendo utilizado. É preciso preocupar-se com os usos políticos da genética, incluindo seu emprego como instrumento retórico de persuasão e, agora, como ferramenta na construção e essencialização de identidades e concepções raciais. Além disso, esses testes reforçam o mito do DNA como a molécula da vida e reintroduzem a metáfora do programa genético, renovando o determinismo genético com uma nova roupagem. Como já apontavam Ricardo Ventura Santos, Maria Cátira Bortolini e Marcos Chor Maio (2006, p.34) no começo do século XX, ainda estamos caminhando no fio da navalha, pois resultados genéticos podem ser reinterpretados culturalmente e influenciar perspectivas raciais, ao mesmo tempo que ameaçam reeditar e reforçar concepções biológicas de raça.

No livro *Permanent markers*, Sarah Abel desafia a visão simplista dos testes de DNA como uma compreensão definitiva da identidade, destacando a multiplicidade das relações entre genética e identidade. Oferece uma análise ampla das teorias contemporâneas sobre raça e genética, criticando o papel histórico da ciência na construção dessa concepção e defendendo uma abordagem biocultural. Abel integra observações teóricas de campos diversos, como genética, antropologia e filosofia, com narrativas pessoais e entrevistas realizadas durante a pesquisa. A leitura é fluida e envolvente, contribuindo significativamente para os estudos de raça e genética, sendo recomendada para acadêmicos, profissionais e ativistas interessados na interseção entre ciência, identidade e poder. Por tudo isso, sua tradução é tarefa fundamental.
